# Hand hygiene compliance in a Brazilian COVID-19 unit: the impact of moments and contact precautions

**DOI:** 10.1186/s13756-023-01356-3

**Published:** 2024-01-22

**Authors:** Marília Duarte Valim, Jéssica Regina Rossetto, Juliano Bortolini, Loreen Herwaldt

**Affiliations:** 1 Nursing Department, Federal University of Mato Grosso, Cuiabá, Mato Grosso Brazil; 2 Statistics Department, Federal University of Mato Grosso, Cuiabá, Mato Grosso Brazil; 3 Department of Internal Medicine, Carver College of Medicine, University of Iowa, Iowa City, IA USA

**Keywords:** Hand hygiene, Cross infection, Compliance, Intensive care units, Patient safety, COVID-19

## Abstract

**Background:**

Healthcare-associated infections are among the most common complications during hospitalization. These infections increase morbidity and mortality and they increase length of hospital stay and the cost of healthcare. The aims of our study were to monitor hand hygiene (HH) compliance, HH technique quality and factors related to HH practice among health professionals in a COVID-19 Intensive Care Unit (ICU).

**Methods:**

An observational, prospective study. Between September and December 2021, we observed 69 healthcare professionals in an eight-bed ICU for patients with COVID-19 in midwestern Brazil. We used the WHO observation form to collect data. The dependent variable was HH compliance and independent variables were professional category, sex, HH quality (3-step technique for at least 15 s), number of HH opportunities observed, observation shift and inappropriate glove use.

**Results:**

We observed 1185 HH opportunities. The overall compliance rate was 26.4%, but only 6.5% were performed with the correct 3-step technique for the minimum time. HH compliance was considerably lower for moments “before” tasks (6.7%; 95% CI 4.8%, 9.2%) compared with moments “after” tasks (43.8%; 95% CI 39.9%, 47.8%). The logistic model found that inappropriate glove use, night shift and physicians (*p* < 0.001) were associated with low HH compliance. The infrastructure analysis found that the unit had an insufficient number of alcohol-based handrub (ABHR) dispensers at the point of care and that the mechanism for activating them was poorly designed.

**Conclusions:**

HH compliance was very low. Inappropriate glove use was associated with low compliance and the unit’s infrastructure did not support good HH practice. The fact that healthcare professionals were more likely to do HH after tasks, suggests that they use HH to protect themselves rather than the patients. Adequate infrastructure and ongoing health education with a focus on HH while caring for patients in contact precautions are essential for improving HH compliance and patient safety.

**Supplementary Information:**

The online version contains supplementary material available at 10.1186/s13756-023-01356-3.

## Backgound

Issues related to patient safety constitute a global health concern. Healthcare-associated infections (HAIs) are among the most common complications during hospitalization. These infections increase morbidity and mortality and they increase length of hospital stay and the cost of healthcare [33].

An annual report from the European Center for Disease Prevention and Control (ECDC) stated that about 8% of patients hospitalized in an Intensive Care Unit (ICU) for more than 2 days acquired at least one HAI [10]. In contrast, Machado et al. [20] recently identified 136 infected patients hospitalized in intensive care units (ICUs) in 15 Brazilian public hospitals of whom 106 (77.94%) contracted at least one HAI in the ICU.

Hand hygiene (HH) is a low-cost and highly effective measure for preventing pathogen spread and for reducing HAIs [2]. For example, Xun et al. found that higher HH compliance rates were associated with lower a risk of pathogen transmission [47]. Schreiber et al. found that multimodal interventions could reduce HAI rates 35 to 55% regardless of a country’s development index [30].

The World Health Organization (WHO) developed and recommends using a “Multimodal Hand Hygiene Improvement Strategy” [41], which includes five complementary and interdependent intervention components: (1) system change; (2) training and education; (3) evaluation and feedback; (4) reminders in the workplace; and (5) institutional safety climate. Studies, including some done during the COVID-19 pandemic, have shown that this strategy is an effective and adaptable method for improving HH compliance rates and reducing transmission of HAI-causing pathogens [3, 4, 21, 37].

Nevertheless, HH compliance among healthcare professionals caring for patients with COVID-19 remains lower than recommended. These findings may be related to work overload and stress, physical discomfort caused by constant use of personal protective equipment (PPE) [3, 44], inappropriate glove use when caring for patients in contact precautions [29], inadequate infrastructure and poor safety climate [14].

To ensure that HH is effective, infection prevention programs should monitor compliance rates and HH quality, including whether healthcare professionals performed all recommended steps for the correct time [24]. Recent studies have shown that a 3-step technique—(1) cover all surfaces of the hands, (2) rotational rubbing of the fingertips in the palm of the opposite hand; and (3) rotational rubbing of both thumbs—is as effective as the WHO 6-step technique [35, 36]. Likewise, recent studies have shown that 15 s is as effective as 30 s in reducing bacterial counts on the hands when alcoholic preparations are used [13, 34].

Widmer and Dangel [45] demonstrated that healthcare professionals, including those who were highly trained, had major deficiencies in their HH technique. Subsequently, Widmer reported that compliance with all 6 HH steps was 13.4% and that 83–90% of the nurses and 95–97% of the physicians observed missed the steps involving the fingertips and the thumb [46]. More recently, Stadler and Tschudin-Sutter [32] reviewed the literature on interventions to decrease HAIs by increasing HH compliance. They reported that rigorous and systematic education, better HH surveillance systems, reducing the time required for HH and reducing the number of HH steps can improve compliance and decrease HAI rates. Given these findings, we need good data on both HH compliance and on healthcare professionals’ HH technique including the HH steps completed and the time spent doing HH.

### Objectives

This study aimed to monitor HH compliance and HH technique and to identify factors affecting HH practice among healthcare professionals working in a COVID-19 ICU.

## Method

### Study design

We conducted an observational, prospective study. We report our results according to the Strengthening the Reporting of Observational studies in Epidemiology (STROBE) checklist [40].

### Participants and setting

The study took place in an eight-bed ICU for patients with COVID-19 at a medium-sized 116 hospital beds, tertiary care university hospital in midwestern Brazil. The unit opened in April 2020 and closed in December 2021. We observed practice from August to December 2021. 69 of 74 (93.2%) healthcare professionals who performed patient care related activities agreed to participate in the study and signed the consent document.

Sixty-nine healthcare professionals, including 34 nursing assistants, 14 nurses, 13 physicians and 8 physiotherapists, participated in the study. We included healthcare professionals who performed patient care-related activities and we excluded staff members who performed administrative functions exclusively.

### Variables

The dependent variable was HH compliance based on WHO indicated moments for HH (OPAS; [7, 44]). Independent variables were professional category, sex, HH quality (steps performed and time spent), number of HH opportunities observed, observation shift, glove use and unit HH infrastructure.

### Measurement

Participants completed a questionnaire with questions about their age, sex, education level and professional category. We used the WHO observation form, which is a validated instrument, to collect HH compliance data [42]. The form was modified during the COVID-19 pandemic to include HH done before PPE donning and doffing [44]. Thus, we monitored the following seven HH moments: (1) Before touching a patient, (2) Before clean/aseptic procedures; (3) After body fluid exposure/risk; (4) After touching a patient; (5) After touching a patient’s surroundings; (6) Before putting on PPE; (7) After removing PPE.

The WHO observation form allows observers to record the total number of HH opportunities, the number of times healthcare professionals did HH, if healthcare professionals wore gloves, if they performed HH, and whether they used an alcohol product or soap and water when they performed HH. On the basis of the WHO guidelines, we observed practice in 20-min blocks, but we extended some sessions depending on the procedures performed (see Additional file 1).

We used a WHO questionnaire to assess the unit’s infrastructure for HH. The instrument has 27 questions about the physical resources for HH on a unit, such as: water availability; number of beds; number of sinks equipped with water, soap and paper towels; number of alcohol dispensers within reach; whether the dispensers are in good working condition and are supplied with alcohol; whether HH posters are present on the unit and where they are located; whether procedure gloves are available; number of medical professionals, nurses and nursing assistants on each unit, and whether the institution provides HH training and audits HH compliance [43].

### Bias

The principal investigator used practical simulations based on the WHO Multimodal Hand Hygiene Improvement Strategy to train the undergraduate student and the graduate student who directly observed healthcare professionals’ HH. To assess whether the two observers were trained adequately and assess their level of agreement, they simultaneously assessed 42 HH opportunities and we calculated the Kappa coefficient. In addition, they observed seven healthcare professionals on a medical ward for patients with COVID-19 during three 20-min observation sessions. The principal investigator was the gold standard. The Kappa coefficient was 0.84, and thus was classified as near-perfect agreement [17]. These observations were not included in the study itself.

To avoid bias, the observations were conducted during all work shifts (morning, afternoon and night), and on holidays and weekends. To minimize the Hawthorne effect, healthcare professionals received information on the study and signed the research Informed Consent Form at least 1 month before they were monitored.

To assess HH technique, we used the 3-steps described by Tschudin-Sutter et al.: (1) covering all surfaces of the hands; (2) rotational rubbing of the fingertips in the palm of the opposite hand; and (3) rotational rubbing of both thumbs [35, 36]. We used a digital chronometer, which was tested and approved by the Brazilian National Institute of Metrology, Quality and Technology (INMETRO—*Instituto Nacional de Metrologia, Qualidade e Tecnologia*), to measure HH duration. We defined the optimal HH duration as 15 s when using alcoholic preparations and 40 to 60 s when doing HH with soap and water [13].

### Statistical methods

We calculated healthcare professionals’ HH compliance rates by dividing the number of HH opportunities at which healthcare providers performed HH actions by the total number of HH opportunities observed during a session [41]. We calculated compliance with appropriate HH technique as the number of actions performed with appropriate technique divided by the number of HH actions performed. We also divided the number of times healthcare professionals wore gloves during missed HH opportunities by the total number of missed HH opportunities to estimate the percent of missed HH opportunities that might be explained by glove use.

We calculated confidence intervals for proportions, did the bivariable binomial test for difference between two proportions and did multiple logistic regression to assess the association of HH compliance with professional category, sex, HH quality (steps performed and time spent), total number of HH opportunities (WHO’s HH moments), observation shift, glove use and unit infrastructure for HH. We performed likelihood ratio tests to assess the model’s significance and identify the variables that were significantly associated with HH performance. We calculated odds ratios to assess the effect size of these associations. We used McFadden’s R^2^ to assess the model quality. We defined statistical significance as a p-value of 0.05 or as 95% confidence intervals that did not cross one. We used R software for all statistical analyses.

### Ethical aspects

The Research Ethics Committee approved this study under Opinion 3,545,329. The study received the Certificate of Presentation for Ethical Consideration 75169317.0.0000.5541.

## Results

Sixty-nine healthcare professionals, including 34 (49.27%) nursing assistants, 14 nurses (20.28%), 13 physicians (18.84%) and 8 physiotherapists (11.59%), participated in the study. Of the participants, 48 (69.56%) were female and 21 (30.43%) were male. The mean age for participants was 43.44 years (range: 24 to 63 years). Twenty-eight participants (40.57%) had postgraduate specialization and 20 (28.98%) had completed higher education, of which 2 (2.89%) had master’s degrees and one (1.44%) had a PhD. Forty-four (63.76%) participants answered the question about their most recent HH training, of whom 28 (63.63%) had training in 2020, 12 (27.27%) in 2021, 3 (6.81%) in 2019 and 1 (2.27%) before 2019.

We observed 1185 HH opportunities. The overall HH compliance rate was 26.50% (314), but only 19 (6.50%) actions included all 3 recommended steps and met the minimum time requirement. Only 14.3% (95% CI 0.106, 0.187) of HH actions were done with an alcohol-based hand rub and 85.7% (95% CI 0.813, 0.894) were done with soap and water.

Table 1 presents the data on HH opportunities, general HH compliance rates, and compliance with adequate technique by professional category, sex and WHO HH moments. The moments “before touching a patient” and “before clean/aseptic procedures” had the lowest compliance rates (3.93% and 1.88%, respectively) and none of these HH actions complied with the recommended technique. All 314 HH actions complied with the first step, “covering all surfaces of the hands.” Compliance with the second step, “rotational rubbing of the fingertips in the palm of the alternate hand”, was only 13.05% (41/314) and compliance with the third step, “rotational rubbing of both thumbs,” was 35.66% (112/314). The mean time spent on HH with alcoholic preparations was 9.84 s (SD 6.83; range: 3 to 42 s). The mean time for hand washing with soap and water was 24.48 s (SD 15.87; range: 4 to 95 s).

**Table 1:** Compliance with hand hygiene and appropriate technique, by profession, sex and hand hygiene moments

Variables	Opportunities	Actions	Compliance (%)	95% CI	Adequate technique	Compliance with adequate technique (%)	95% CI
*Professional category*							
Nurses	246	79	32.1	(26.3–38.0)	16	20.3	(11.4–29.1)
Nursing assistants	694	166	23.9	(20.8–27.1)	2	1.2	(0.0–2.9)
Physiotherapists	137	40	29.2	(21.6–36.8)	0	0.0	(0.0–0.0)
Physicians	108	29	26.9	(18.5–35.2)	1	3.5	(0.0–10.1)
*Sex*							
Male	378	110	29.1	(24.5–33.7)	6	5.5	(1.2–9.7)
Female	807	204	25.3	(22.3–28.3)	13	6.4	(3.0–9.7)
*Moments*							
Before touching a patient	229	9	3.9	(1.4–6.5)	0	0.0	(0.0–0.0)
Before clean/aseptic procedures	160	3	1.9	(0.0–4.0)	0	0.0	(0.0–0.0)
After body fluid exposure/risk	201	44	21.9	(16.2–27.6)	1	2.3	(0.0–6.7)
After touching a patient	178	59	33.2	(26.2–40.1)	5	8.5	(1.4–15.6)
After touching patient surroundings	86	33	38.4	(28.1–48.7)	2	6.1	(0.0–14.2)
Before putting on PPE	164	25	15.2	(9.7–20.8)	0	0.0	(0.0–0.0)
After removing PPE	167	141	84.4	(78.9–89.9)	11	7.8	(3.4–12.2)
*Shift*							
Morning	614	187	30.5	(26.8–31.1)	12	6.4	(2.9–9.9)
Afternoon	314	76	24.2	(19.5–28.9)	1	1.3	(0–3.9)
Night	257	51	19.8	(15.0–24.7)	6	11.8	(2.9–20.6)
*Glove use*							
No	776	313	40.3	(36.9–43.8)	19	6.1	(3.4–8.7)
Yes	409	1	0.2	(0–0.7)	0	0	(0–0)
Total	1185	314	26.5	(24.0–29.0)	19	6.1	(3.4–8.7)

CI, confidence interval

Table 2 shows the total HH opportunities, missed HH opportunities and frequency of glove use during missed HH opportunities. Of the 707 missed HH opportunities, 409 (46.96%) were associated with inappropriate glove use. Inappropriate glove use during missed HH opportunities was observed most frequently for the moments “before clean/aseptic procedures,” “before touching a patient” and “after body fluid exposure risk”. HH compliance was considerably lower for moments “before” tasks (6.7%; 95% CI 4.8%, 9.2%) compared with moments “after” tasks (43.8%; 95% CI 39.9%, 47.8%). The bivariable binomial analysis found that healthcare professionals were significantly less likely to do HH “before clean/aseptic procedures” and “before touching a patient” (3.1%; 95% CI 1.7%, 5.5%) then they were to do HH “after body fluid exposure risk” and “after touching a patient” (27.2%; 95% CI 22.8%, 32%).

**Table 2:** Opportunities, missed hand hygiene opportunities glove use, proportions and confidence interval according to moments

Moments	Opportunities	Missed actions n (%)	Frequency of glove use	Proportion of glove use during missed HH actions (%) (CI)
1-Before touching a patient	229	220 (96.1)	132	60.0
				(53.5–66.5)
2-Before clean/aseptic procedures	160	158 (88.8)	135	86.0
				(80.6–91.4)
3-After body fluid exposure/risk	201	157 (78.1)	95	60.5
				(52.9–68.2)
4-After touching a patient	178	119 (66.9)	34	28.57
				(20.5–36.7)
5-After touching patient surroundings	86	53 (61.3)	8	15.09
				(5.5–24.7)
6-Before putting on PPE	164	139 (84.8)	NA	
7-After removing PPE	167	26 (15.6)	NA	

CI, confidence interval; NA, not applicable

The logistic model for HH compliance rates (Table 3) found that nurses, physiotherapists, and nursing assistants were significantly more likely to do HH than were physicians. HH compliance was significantly worse during the afternoon and night than during the morning and significantly worse during the night than during the afternoon. Moreover, HH compliance was significantly better “after touching a patient” (OR 7.21; 95% CI 3.34, 15.58; *p* < 0.001), “after body fluid exposure/risk” (OR 6.49; 95% CI 2.93, 14.37; *p* < 0.001), “after touching patient surroundings” (OR 7.58; 95% CI 3.3, 17.39; *p* < 0.001) and “after removing PPE” (OR 63.35; 95% CI 27.76, 144.61; *p* < 0.001) compared with “before touching a patient.” Healthcare professionals’ HH compliance was significantly lower when they wore gloves than when they did not. Healthcare professional’s sex was not associated with HH compliance. McFadden’s R^2^ confirmed the model’s findings that doing HH at the recommended HH moments, the observation period and glove use were significantly associated with HH compliance (*p* < 0.001; McFadden’s R^2^ = 0.422).

**Table 3:** Logistic regression related to compliance by moments, glove use, profession, sex and observation period

Variables	Odds ratio	95% confidence interval		*p*
		Lower limit	Upper limit	
Intercept	0.18	0.08	0.41	< 0.001
*Moments*				
1-Before touching a patient (reference)	1			
2-Before clean/aseptic procedures	1.23	0.30	4.97	0.771
3-After body fluid exposure/risk	6.49	2.93	14.37	< 0.001
4-After touching a patient	7.21	3.34	15.58	< 0.001
5-After touching patients’ surroundings	7.58	3.30	17.39	< 0.001
6-Before donning PPE	1.94	0.85	4.39	0.113
7-After doffing PPE	63.35	27.76	144.61	< 0.001
*Glove use*				
No (reference)	1			
Yes	0.01	0.00	0.06	< 0.001
*Professional category*				
Physician (reference)	1			
Nurse	2.67	1.34	5.34	0.005
Physiotherapists	2.26	1.06	4.83	0.036
Nursing assistants	1.95	1.04	3.67	0.037
*Sex*				
Male (reference)	1			
Female	0.93	0.63	1.35	0.689
*Shift*				
Morning (reference)	1			
Afternoon–morning	0.76	0.50	1.15	0.187
Night–morning	0.36	0.22	0.57	< 0.001
Night–afternoon	0.47	0.28	0.80	0.006

The study ICU had 6 sinks with faucets that were hand activated, one of which was at the entrance to the isolation room, four were between beds and one in the common area, close to the medication preparation space. The trash bins were pedal-operated, but pedals malfunctioned, forcing healthcare professionals to lift the lids with their hands when disposing paper towels used to dry their hands.

Although there were eight beds in the ICU, ABHR preparations were available by only six of the beds, three of which were difficult to access because the dispensers were located behind patients’ beds or equipment, such as an infusion pumps, mechanical respirators, IV stands, chairs, or emergency carts, rather than at the point of care. Moreover, some soap or alcoholic preparation dispensers were empty and all ABHR dispensers were activated by pressing upwards with the fingertips, which was very awkward.

## Discussion

HH compliance was low and the HH technique was poor and did not maximize the antiseptic effect of the alcohol hand rub. Low HH compliance is a problem worldwide [16], and previous studies have shown that most healthcare professionals do not perform the rotational rubbing of both thumbs and do not spend the minimum time necessary for proper antiseptic action [11, 31]. We previously observed 60 nursing professionals care for patients in a Brazilian COVID-19 unit [38]. We found that, despite the low HH compliance, only 13.3% of healthcare professionals performed the correct 3-step HH technique. Although compliance with the first step “covering all surfaces of the hands” was 100%, only 20 (52.6%) of the nursing professionals performed the second step “rotational rubbing of the fingertips in the palm of the opposite hand” and only by 12 (31.6%) performed the third step “rotational rubbing of both thumbs”.

The logistic model found that compliance with the moments “before” tasks was considerably lower than the moments “after” tasks, which is consistent with results of prior studies [9]. In addition, other studies have found that healthcare professionals did HH more often after contaminated tasks than before clean procedures or aseptic tasks [8, 9, 48, 49]. In fact, Bezerra et al. monitored 3025 HH opportunities and found that HH compliance was highest “after body fluid exposure risk” (60.80%) and “after touching a patient” (53.45%). Like Chang et al. [9], they concluded that healthcare professionals tend to protect themselves from exposure to potentially contaminated biological material and neglect patient safety [6]. Stadler and Tschudin-Sutter suggested that healthcare providers do not perceive HH to be a communal responsibility and do not acknowledge that HH is a “duty of care towards their patient.” Rather they view HH “as duty of care towards themselves” [32].

In our study, the highest HH compliance was observed after doffing PPE; however, a recent study found that only 35% of healthcare professionals knew the correct order for donning and doffing PPE [25]. Moreover, the greatest risk for hand contamination occurred when healthcare professionals removed their gloves before they removed their protective aprons (*p* = 0.025) [22]. We found that healthcare professionals often removed their gloves followed by their aprons and then sanitized their hands rather than following the WHO guidance to sanitize their hands immediately after removing their gloves.

Hospitalized patients with confirmed or suspected COVID-19 should be placed in contact and droplet precautions. However, glove use does not replace HH [7, 9]. Studies of healthcare professionals’ HH complicance when caring for patients in contact precautions have demonstrated that glove use is associated with both decreased HH and an increased risk of pathogen transmission [5, 23]. We found that inappropriate glove use was associated with lower HH compliance in our COVID-19 ICU, which is similar to the results of prior studies [9, 15]. In addition, studies in both developed [1, 9, 29] and developing countries [6] have found that inappropriate glove use was a strong predictor for non-compliance with HH. Inappropriate glove use may have been exaccerbated due to the requirement to use contact precautions while caring for patients with COVID-19, which may help explain the high rate of inappropriate glove use in our study.

We observed that many nursing assistants double gloved while performing procedures on a patient, which led to non-compliance with HH, especially at the moments before touching a patient and before clean/aseptic procedures. Similar to Rio et al. [26], we observed healthcare professionals wearing the same pair of gloves while caring for different patients in the unit or wearing contaminated gloves while performing different tasks, including those that should be done aseptically, on a patient. This practice contradicts Brazilian guidelines for COVID-19 prevention and control [7].

Of note, Lindberg et al. [18] found that healthcare professionals touched many surfaces in patients’ rooms with contaminated gloves; the most frequently touched items were panels of multi-parameter monitors, switches, infusion pumps and patients’ belongings. Given the propensity of healthcare professionals to use gloves in this way, Vogel et al. assessed disinfecting gloves with an hydroalcoholic solution. They found that cultures from 79.3% of the “disinfected” gloves were negative. On the basis of their findings, they suggested that alcohol-based disinfection of gloves could be a reasonable alternative to current recommendations [39]. To date, WHO has not recommended this practice.

Similar to other studies [9], we found that physicians have the lowest HH compliance,their most frequently missed moments were those before and after touching a patient. In addition, they visited different patients without doing hand hygiene and they frequently used one pair of gloves to provide care to more than one patient.

The HH infrastructure on our COVID-19 ICU was poor, which likely had a negative influence on HH compliance. Some ABHR dispensers were empty and the method for dispensing the hand sanitizer (fingertip pressure) was awkward and difficult. Moreover, many dispensers were located distant from the point of care or were hidden behind or between advanced life support devices. These deficits may help explain why healthcare professionals usually did HH with soap and water rather than with the ABHR.

A good HH infrastructure is the first prerequisite for increasing HH rates and the institution must ensure the appropriate HH supplies are available at the point of care. Higher compliance rates have been achieved by placing ABHR bottles at the point of care and providing washbasins with manual faucets [1, 6]. But inadequate supplies of ABHR, empty dispensers, poorly located dispensers [28], insufficient or inaccessible sinks undermine efforts to improve HH compliance [8, 27].

Of note, WHO guidelines emphasize the need to provide ABHR as the preferred product for HH. Toward that end WHO states that healthcare facilities must ensure that an adequate supply of ABHR is continuously available at the point of care so that healthcare providers can perform HH at all recommended moments. In addition, ABHR has a broad antimicrobial spectrum, is highly effective and is kinder to the skin and takes less time than HH with soap and water, which could help facilitate HH compliance [44].

Our study had several limitations. First, we observed practice in one hospital, which limits the external validity of our findings. In addition, the presence of observers may have affected healthcare professionals’ behavior, which may have affected our results [12]. To reduce this effect, we observed practice at different times, and we observed different healthcare professionals.

## Conclusion

HH compliance was low in our COVID-19 ICU as was compliance with all HH steps and the time required for adequate antisepsis. Given that their HH complicance was significantly better after tasks than before, healthcare professionals appeared to be more concerned about protecting themselves than with preventing HAIs. Our finding that inappropriate glove use, profession (i.e., physicians), night shift and poor HH infrastructure on the unit negatively influenced HH compliance suggest that healthcare facilities must provide adequate HH infrastructure and training in good HH technique and correct glove use, including appropriate donning/doffing, if they want to improve HH compliance and efficacy and thereby improve patient safety. Moreover, our study highlights the need for urgent improvements in HH supplies and HH infrastructure, as they are essential components of the multimodal strategy, without which compliance cannot be achieved or sustained. Furthermore, our study highlighted the importance of implementing multimodal strategies—providing an adequate number of well-stocked, functioning hand sanitizer dispensers and educating staff about correct glove use when caring for patients who are in contact precautions—so that patient care is performed safely and the risk of healthcare-associated infections is minimized.

### Supplementary Information

**Additional file 1.** WHO Observation Form.

## Data Availability

The datasets used and/or analyzed during the current study are available from the corresponding author on reasonable request. The Dean of Postgraduate Students, Federal University of Mato Grosso (Brazil). The Coordination of Superior Level Staff Improvement.

## Abbreviations

HH: Hand hygiene
HAIs: Healthcare-associated infections
ICU: Intensive care unit
ECDC: European Center for Disease Prevention and Control
WHO: World Health Organization
PPE: Personal protective equipment
STROBE: Strengthening the reporting of observational studies in epidemiology
N: Number of participants
CI: Confidence interval
NA: Not applicable
SD: Standard deviation
