# Supplementary material for: Hand hygiene compliance in a Brazilian COVID-19 unit: the impact of moments and contact precautions
Source: Antimicrob Resist Infect Control. 2024 Jan 22;13:7. doi: 10.1186/s13756-023-01356-3 (PMC10801978; doi:10.1186/s13756-023-01356-3)
Supplement: Supplementary file 1 — Additional file 1. WHO Observation Form. [file 13756_2023_1356_MOESM1_ESM.docx]

**Additional File 1** – Who observation form.
